# CPAP with variable flow is comparable with Bubble CPAP in preterm infants

**DOI:** 10.1186/cc10184

**Published:** 2011-06-22

**Authors:** CM Rebello, ACZ Yagui, LA Vale, LB Haddad, C Prado, FS Rossi, AD Deutsch

**Affiliations:** 1 Hospital Israelita Albert Einstein, São Paulo - SP, Brazil

## Background

nCPAP has many benefits to treat respiratory distress in the newborn. It has been shown that in devices with variable flow, nCPAP reduces work of breathing and increases lung recruitment, compared with continuous flow; nevertheless, there are few randomized controlled trials comparing these different CPAP apparatuses regarding respiratory outcomes.

## Objective

To evaluate the efficacy of nasal CPAP using a device with variable flow or Bubble CPAP, regarding CPAP failure, occurrence of air leaks, total CPAP time and main complications of prematurity.

## Methods

A randomized clinical trial. Newborns admitted to the Hospital Israelita Albert Einstein's NICU (São Paulo, Brazil) with birth weight ≥1,000 g, without previous mechanical ventilation and with respiratory distress requiring nCPAP were randomized into two study groups: Variable Flow (Servo-*I*; Siemens Elema Inc., Sweden) or Bubble CPAP (Fisher and Paykel Healthcare, Auckland, New Zealand). Both groups used the same interface (BC 161; Fisher and Paykel Healthcare), with a target SatO_2 _of 88 to 94%. Gestational age, birth weight, Apgar 5 minutes, diagnosis of respiratory distress, CPAP failure, the main complications of prematurity and total CPAP and oxygen time were recorded. Continuous variables were analyzed by Student *t *test, categorical variables were analyzed by Fisher's exact test. The significance level was set at *P *= 0.05.

## Results

A total of 40 infants were randomized. One baby was excluded from the Variable Flow Group because we were obligated to change the nasal prong interface due to the development of nasal injury and damage to the septal mucosa. There were no differences between groups regarding birth weight (Variable Flow: *n *= 19, 2,602 ± 585 g; Bubble CPAP: *n *= 20, 2,518 ± 598 g; *P *= 0.663); gestational age (35.8 ± 0.5 weeks and 35.7 ± 0.4 weeks; *P *= 0.863); gender (male: 68.4% and 70.0%; *P *= 0.915); Apgar5 (9.4 ± 0.6 and 9.6 ± 1.0; *P *= 0.246); prenatal steroids (31.6% and 10.0%; *P *= 0.127); time for CPAP installation (120/90/203 minutes and 135/50/225 minutes; *P *= 0.978); CPAP failure (21.1% and 20.0%; *P *= 1.000); air leak syndrome (10.5% and 5.0%; *P *= 0.605); total CPAP time (22.0/8.00/31.00 hours and 22.0/6.00/32.00 hours; *P *= 0.822); and total oxygen time (24.00/7.00/85.00 hours and 21.00/9.50/66.75 hours; *P *= 0.779). Values are mean ± SD or percentage or median/interquartile ranges (for time for CPAP installation, total CPAP and total oxygen time).

## Conclusion

In this small randomized clinical trial the use of a device with variable flow was comparable with Bubble CPAP regarding the occurrence of the main variables analyzed.

